# Cognitive Modulation of Psychophysical, Respiratory and Autonomic Responses to Cold Pressor Test

**DOI:** 10.1371/journal.pone.0075023

**Published:** 2013-10-09

**Authors:** Enrica L. Santarcangelo, Giulia Paoletti, Iacopo Chiavacci, Carlo Palombo, Giancarlo Carli, Maurizio Varanini

**Affiliations:** 1 Department of Translational Research and New Technologies in Medicine and Surgery, University of Pisa, Pisa, Italy; 2 Department of Surgery, University of Pisa, Pisa, Italy; 3 Department of Physiology, University of Siena, Siena, Italy; 4 Institute of Clinical Physiology, National Council of Research, Pisa, Italy; University of Bologna, Italy

## Abstract

In healthy subjects with high hypnotisability (*highs*) under hypnosis, subjectively effective suggestions for analgesia abolish the increases in blood pressure associated with cold pressor test (cpt) by reducing the peripheral vascular resistance. The aim of the present study was to investigate the effects of the suggestions of analgesia on the responses to cpt in healthy *highs (*n = 22) and in low hypnotisable participants (*lows*, n = 22) out of hypnosis. Cpt was administered without (CPT) and with suggestions for analgesia (CPT+AN). Psychophysical (pain intensity, pain threshold, cpt duration (time of immersion) and pain tolerance, defined as the difference between cpt duration and pain threshold), respiratory (amplitude and frequency) and autonomic variables (tonic skin conductance, mean RR interval (RR = 1/heart rate), blood pressure, skin blood flow) were studied. The suggestions for analgesia increased cpt duration and RR in both groups, but decreased pain intensity and enhanced pain threshold only in *highs*; in both groups they did not modulate systolic blood pressure, tonic skin conductance and skin blood flow; thus, increased parasympathetic activity appears responsible for the heart rate reduction induced by suggestions in both groups. In conclusion, our findings show that suggestions modulate pain experience differentially in *highs* and *lows,* and are partially effective also in *lows.* We hypothesize that the mechanisms responsible for the efficacy of suggestions in healthy *lows* may be involved also in their efficacy in chronic pain patients with low hypnotisability.

## Introduction

The autonomic activity is important in pain modulation as it is monitored at cortical level and contributes to the construction of the individual experience [1], [2].

Cold pressor test is a good tool to investigate the autonomic correlates of pain modulation because it shifts the autonomic state toward a sympathetic prevalence in the majority of the general population [3], [4] and, thus, its employment reduces the probability of negative findings depending on the large variability of the responses to nociceptive stimulations [5]. Such variability may have masked the autonomic correlates of pain modulation in earlier studies performed in subjects with high (*highs*) and low (*lows*) hypnotisability [6], [7], [8].

Hypnotic suggestions for analgesia administered during cold pressor test (cpt) are known to be subjectively effective in healthy *highs* [9], [10], [11] and, to some extent, also in subjects with medium hypnotisability scores (*mediums*) [11]. In *highs*, suggestions modulate the autonomic correlates of pain experience by reducing the increase in the peripheral vascular resistance induced by cpt. In contrast, no significant effects of cold pressor test on respiratory patterns have been reported [12], [13], although respiratory frequency and amplitude are responsive to cognitive-emotional states [14], [15] and have shown hypnotizability-related responses to other nociceptive stimulations [8].

After hypnotic induction, subjective beneficial effects of suggestions administered during various nociceptive stimulations have been reported also in *lows* [16], [17], [18], [19], although many studies comparing *highs* and *lows* have shown significantly more pronounced effects in *highs* [7], [8], [20], [21], [22], [23]. Nonetheless, increasing evidence shows that suggestions modulate pain experience both in and out of hypnosis [6], [7], [8], [18], [24], [25]. Thus, the aim of the present study was to investigate the psychophysical, respiratory and autonomic correlates of pain modulation induced by non-hypnotic suggestions of analgesia in *highs* and *lows* undergoing cold pressor test.

## Methods

### Subjects

The experimental protocol was approved by the Ethics Committee of the University of Pisa (n.3180, 2011). Participants signed an informed consent following the rules of the Declaration of Helsinki and describing the experimental procedure, but not the aims of the experiment. Fourty-four healthy volunteers (age, mean±SD: 21±1.7 yrs) were selected according to their hypnotic susceptibility through the *Italian* version of the Stanford Hypnotic Susceptibility Scale, form C [26] among 280 students of the University of Pisa. They were divided in two groups: 22 highly (*highs*, score ≥9/12, 11 females) and 22 low hypnotizable individuals (*lows*, score ≤3/12, 10 females). The percentage of *highs* and *lows* found among participants was consistent with the commonly observed hypnotizability distributions [26], [27], [28]. Cardiovascular disease and any other systemic disease were ruled out by detailed clinical history and routine biochemistry. None of the subjects reported cardiovascular risk factors (systemic hypertension, diabetes mellitus, hypercholesterolemia, smoking) and previous experience of relaxation techniques. All of them had normal resting electrocardiogram (ECG) and blood pressure. On the day of the hypnotic assessment, they completed questionnaires concerning trait anxiety (STAI-Y2, State-Trait Anxiety Scale), pain coping strategies (BPCI) and the ability of absorption in cognitive activities (TAS, Tellegen Absorption Scale). In order to minimize the possible effects of the expectation of hypnosis, soon after hypnotic assessment participants were informed that no further hypnotic induction was included in the experimental procedure.

### Experimental Procedure

The experimental session took place at least 1 month after hypnotic assessment and was carried out between 2.00 and 4.00 p.m., at least 4 hours after the latest light meal and 6 hours after the latest caffeine containing beverages, in a semi-darkened, sound-attenuated and temperature-controlled room (20–25°C). Females were tested during the second week after their last menses. Subjects were invited to sit in a comfortable arm-chair; the experiment started approximately 10 minutes (min) later, after sensors placement, stabilization of autonomic parameters and familiarization with the experimental setting. Respirogram, skin conductance (SC), electrocardiogram (ECG), non-invasive photo-plethysmographic finger pulse (PP) and skin blood flow (SBF) were recorded after eye closure during resting (basal: B1, B2, duration = 5 min) and nociceptive stimulation conditions elicited by cold pressor test in the absence (CPT) and in the presence of suggestions for analgesia (CPT+AN). The two sequences (B1- CPT, B2-CPT+AN) were randomly administered among subjects. The suggestions for analgesia were administered throughout the CPT+AN condition.

The cold pressor test was performed by immersion of the left hand in icy cold water (0°–1°) up to the wrist. No circulating pump was used. The test was terminated as soon as the subjects reported unbearable pain (*cpt* duration, sec), and interrupted at min 4 in the subjects not reporting unbearable pain yet. Such large variability in cpt duration had been already described and it had been shown that subjects can be divided in pain tolerant (cpt duration around 5 min) and pain sensitive (cpt duration lower that 60 sec) and that their percentages differ across studies [29]. Before immersion, participants were instructed to declare when they began to feel pain (pain threshold, time from immersion, sec) by saying only “*ora*” (now), in order to avoid signals artefacts. Pain tolerance corresponded to the difference between the cpt total duration (time of immersion, sec) and the pain threshold. This parameter was adopted because, theoretically, the total time of immersion (cpt duration) can change either in the absence or in the presence of changes in pain threshold. In their absence, changes in cpt duration should be attributed to changes in tolerance.

Soon after the cpt termination, participants were asked to score the highest pain intensity perceived during immersion (score: 0–10). Previous studies had shown that, in the general population, the pain perception reported the end of cpt on a Visual Analogue Scale (0 = no pain, 100 = worst imaginable pain) was around 60–70 [10], [11], [30].

The suggestions for analgesia were administered in *Italian*. They consisted of the explicit request to imagine a special glove abolishing pain perception and were associated with instructions for relaxation (*“… you cannot feel pain because the thick glove you are wearing prevents you from feeling it …. the glove’s fabric is thick…you are not disturbed by the cold water at all…the glove protects you…. …thus you can relax at your best …please be quiet and relaxed…more and more relaxed…”*).

### Data Acquisition and Analysis

All signals were acquired at 1 kz sampling rate (National Instruments A/D Converter). The right hand Skin Conductance (SC) was recorded by a Contact Precision Instruments device (Psylab, London, UK) through disposable electrodes placed on the thenar eminence; tonic SC mean values were obtained on consecutive 20 seconds intervals by trimmed averaging, and were expressed in arbitrary units (a.u.). The respiratory signal (respirogram) was recorded through an inductive transducer (CompuMedics Life Systems, Victoria, Australia) wrapped around the chest at the level of the 10th rib. Respiratory cycles were detected and their mean amplitude (RA, difference between the inspiratory and expiratory thorax circumference, arbitrary units) and frequency (RF, breath/min) were obtained by averaging on 20 seconds intervals, which was the minimum time interval allowing a reliable assessment of the respiratory signal (assuming a theoretical frequency of 15 cycles/min). ECG was recorded through 3 M Red Dot Ag/AgCl disposable electrodes placed according to the standard first ECG lead (DI) and amplified by a LACE-Elettronica System amplifier (Pisa, Italy). QRS complexes were automatically detected, artefacts/abnormal beats were discarded and the distances between consecutive R waves of the ECG (RR, instantaneous heart rate = 1/RR) were computed. Finger pulse was monitored through a photopletismograph (Psylab, Contact Precision Instruments, London, UK) with a sensor placed on the third phalanx of the index finger of the right hand. The systolic blood pressure (BP_max_) was derived from the delay between the R wave and the finger Pulse (R to Pulse Transit Time, rPTT) according to the literature recommendation [31]. We could not estimate the mean and the diastolic blood pressure because they are poorly correlated with rPTT and cannot be reliably evaluated without measurement of the pre-ejection period [31]. The microcirculatory skin blood flow (SBF, arbitrary perfusion units), which is sensitive to physical and cognitive stimulation [32], [33], was recorded from the third phalanx of the middle finger of the right hand (probe temperature = 37°) through Laser Doppler flow-metry (PeriFlux PF4, Perimed, Jarfalla, Sweden). The acquired SBF signal was normalized on the mean values of the earliest 10 sec of the first basal condition referred to the values displayed by the Laser Doppler flow-metry instrument. Experiential data and signals have been banked in our lab archive and are available upon request.

### Statistical Analysis

Statistical analyses were performed through the SPSS.15 package after normality assessment (Shapiro-Wilk test). Questionnaires scores were analysed through separate univariate (TAS, STAI-Y2) or multivariate ANOVAs (BPCI). Pain intensity scores, pain threshold, pain tolerance and cpt duration were analysed through repeated measures ANOVA according to a 2 Hypnotizability (*highs, lows*) × 2 Gender (females, males) × 2 Condition (CPT, CPT+AN) design. Unpaired t tests between groups were used for post hoc Bonferroni corrected comparisons.

During CPT most of the subjects had a cpt duration shorter than 60 sec; thus, we analysed the Respiratory Frequency (RF), Respiratory Amplitude (RA), tonic Skin Conductance (SC), RR interval (RR), systolic Blood Pressure (BP_max)_ and maximum Skin Blood Flow (SBF_max)_ across three intervals: the first two 20 sec intervals of cpt (CPT_1_ and CPT_2_ for CPT; CPT +AN_1_ and CPT +AN_2_ for CPT +AN) with respect to the latest 60 seconds of preceding resting periods (b1; b2). A longer basal interval was chosen to buffer the possible effects of spontaneous fluctuations of the studied variables. Repeated measures ANOVAs were applied to each of them according to a 2 Hypnotizability (*highs, lows*) × 2 Gender (females, males) × 2 Condition (CPT, CPT+AN) × 3 Interval (b_1_, cpt_1_, cpt_2_; b_2_, cpt+an_1_,cpt+an_2_) design. The Greenhouse-Geisser ε correction for non- sphericity was applied when requested.

Contrast analysis between intervals and unpaired t tests between groups were used for post hoc Bonferroni corrected comparisons. The degrees of freedom in the respiratory variables, SC and SBF analyses are reduced with respect to the RR and BP_max_ analyses because a few subjects had poor signals in one or both Conditions. Pearson coefficient was evaluated for the correlation between cpt duration and the other psychophysical variables (pain intensity, threshold, tolerance) as well as between psychophysical and autonomic variables within each group to clarify the direction of ANOVA findings. Level of significance was set at p<0.05.

## Results

Three subjects (1 *high*, 2 *lows*) did not complete the experiment owing to fear of pain/distress, thus the analyzed sample consisted of 21 *highs* and 19 *lows*.

### Questionnaires

No significant difference was observed between *highs* and *lows* in trait anxiety scores (STAI-Y2, mean±SD. *Highs,* 43.55±5.22; *lows*, 43.16±5.93), while absorption scores were higher in *highs* (TAS, *highs*, 23.64±4.97; *lows*, 13.84±7.45; F(1,39) = 24.701, p<0.0001). The BPCI did not reveal any significant difference between the two groups, except greater proneness (t(1,39) = 3.339, p<0.002) to use relaxation as a pain coping strategy in *highs* (score: *highs,* 2.75±0.24; *lows*, 1.72±0.23). No gender difference was found.

### Psychophysics

**Figure 1:**
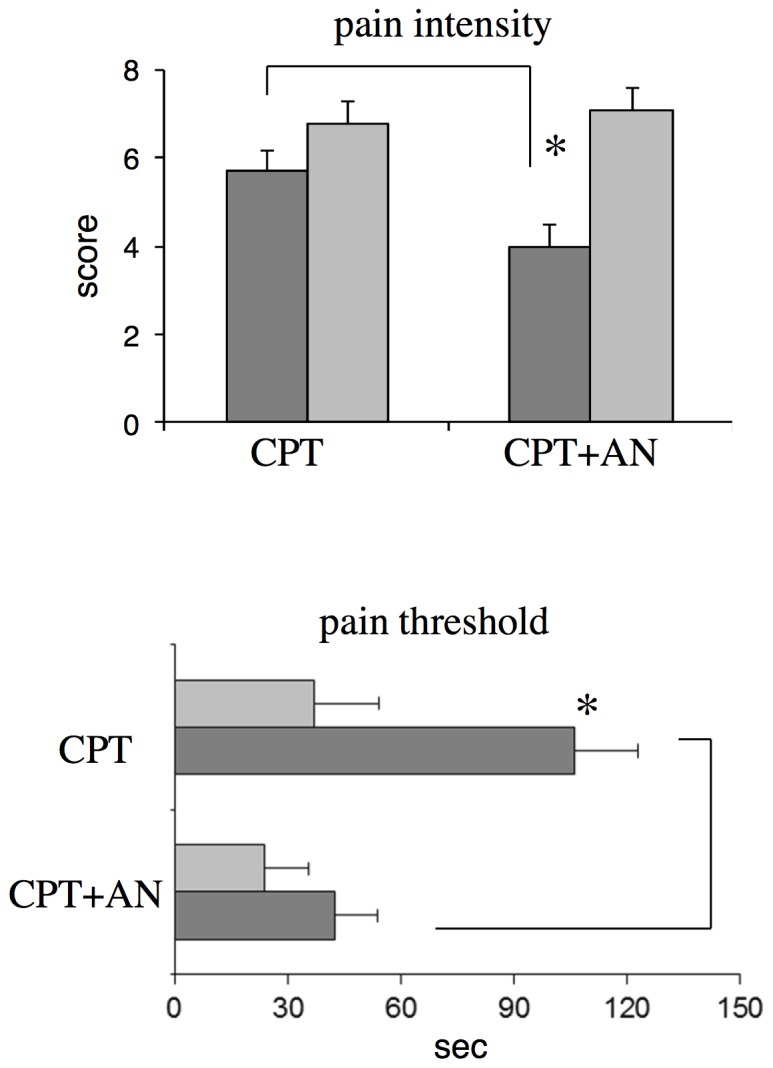
Pain intensity and pain threshold. CPT, CPT+AN: cold pressor test without and with suggestions for analgesia, respectively. Lines, significant differences between conditions; *, significant difference between *highs (*dark bars) and *lows* (light bars).

Pain intensity (Figure 1) exhibited a significant Condition × Hypnotizability interaction (F(1,36) = 7.527, p<0.009) revealing that, during the suggestions of analgesia, *highs* perceived lower pain intensity (about 30%) than during cpt not associated with suggestions (F(1, 20) = 8.682, p = 0.032), whereas *lows* did not show any change; in addition, the pain intensity reported by *highs* at the end of the cpt associated with suggestions was significantly lower than that reported by *lows* (unpaired t test, t (1,38) = 4.453, p = 0.0012).

Pain threshold (Figure 1) exhibited a significant Condition × Hypnotisability interaction (F(1,36) = 6.786, p = 0.013); the suggestions of analgesia increased pain thresholds about three times in *highs* (F(1,20) = 13.298, p = 0.008) and did not change them in *lows*; unpaired t test revealed significantly higher thresholds in *highs* than *lows* only during cpt associated with suggestions (t(1,38) = 2.944, p = 0.020).

Pain tolerance, did not differ between groups and conditions.

**Figure 2:**
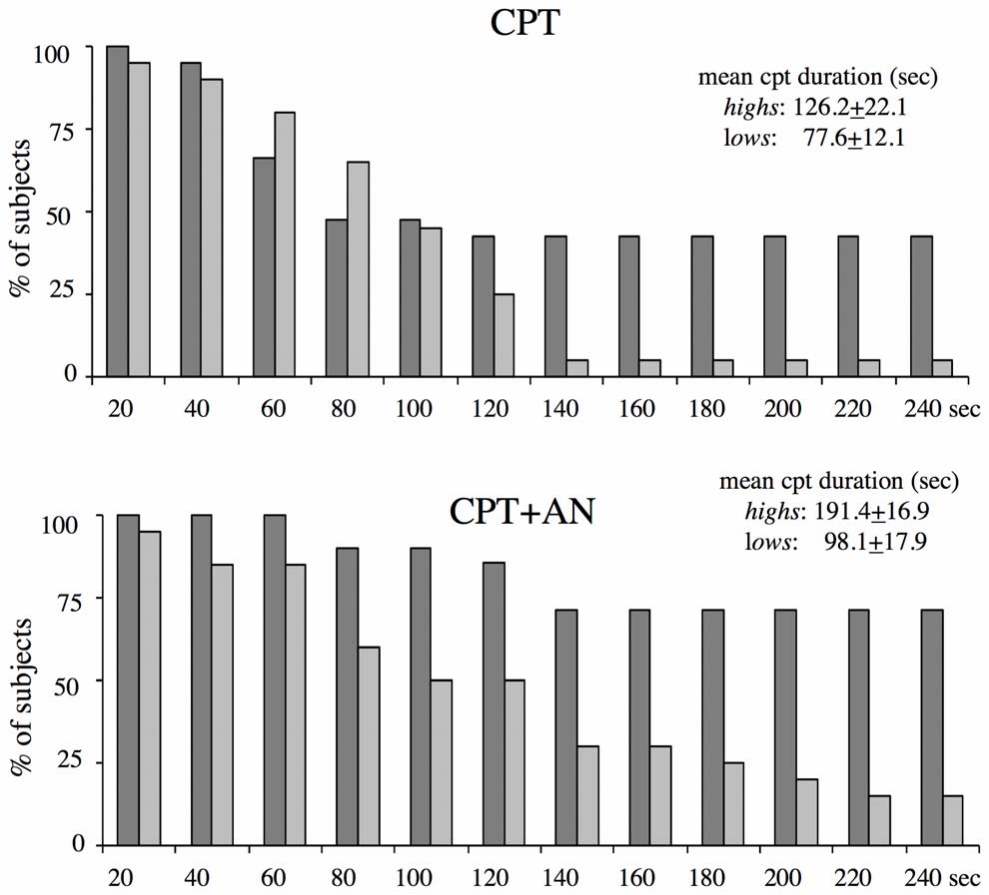
Cold pressor test duration. The suggestions for analgesia (CPT+AN) increased the cpt duration with respect to CPT (cpt without suggestions) in both *highs* (dark bars) and *lows* (light bars) across 20 sec intervals. Bars represent the percentage of *highs* and *lows* keeping left right hand in the icy water for more than 10 sec of each interval.

**Figure 3:**
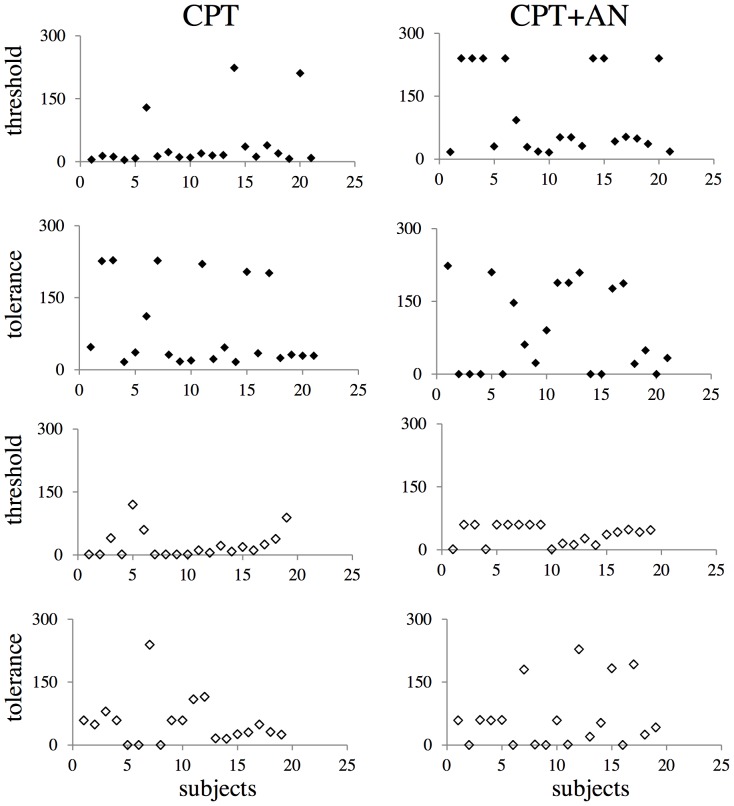
Distribution of pain threshold and tolerance (sec). CPT: cpt without suggestions; CPT+AN: cpt during suggestions of analgesia; *highs*: upper panels (black points); *lows*: lower panels (white points).

The time of immersion (cpt duration) was longer in *high*s than in *lows* (Figure 2) independently of the presence of suggestions (F(1,36) = 10.276, p = 0.001); in both groups it was longer in their presence (F(1,38) = 12.409, p = 0.001). Figure 3 shows the distribution of pain threshold and tolerance in the two groups and conditions.

In the absence of suggestions, cpt duration was positively correlated with both pain threshold (R = .518, p = 0.016) and pain tolerance (R = .775, p = 0.0001) in *highs*, but only with pain tolerance in *lows* (R = .814, p = 0.0001). During suggestions, the time of immersion remained positively correlated only with pain threshold in *highs* (with the same correlation coefficient observed in the absence of suggestions, R = .518, p = 0.016) and maintained its positive correlation with pain tolerance in *lows* (R = .956, p = 0.0001). In both groups TAS scores correlated negatively with the pain intensity reported at the end of the cpt associated with suggestions for analgesia (R = −.688, p = 0.0001), while anxiety did not correlate with any psychophysical variable.

### Breath

No significant effect was found for the respiratory frequency (RF). Post hoc analysis of the significant Interval effect and Hypnotisability × Condition interaction observed for respiratory amplitude (RA) did not reveal significant differences between groups, intervals and conditions (Table 1).

**Table 1:** Summary of respiratory and autonomic effects.

Variable	Effect		Contrast
RA	hypn × condition	F(1, 19) = 5.701, p = 0.027	
		*highs*	ns
		*lows*	ns
		*highs* vs *lows*	ns
	interval	F(2,38) = 5.361, p = 0.022	
		b vs interval 1; b vs interval 2	ns
RF	condition	ns	
	interval	ns	
SC	interval	F(2,50) = 27.929, p = 0.0001	
		interval 1>b	F(1,25) = 27.845, p = 0.0011
		interval 2>b	F(1,25) = 29.748, p = 0.0011
RR	condition	F(1, 74) = 13,453, p = 0.001	CPT<CPT+AN
	interval	F(2, 74) = 46.956, p = 0.0001	
		interval 1<b1	F(1,37) = 60.252, p = 0.0011
		interval 2<b1	F(1,37) = 50.531, p = 0.0011
BP_max_	interval	F(2, 74) = 24.460, p = 0.0001	
		interval 1>b	F(1,37) = 41.738, p = 0.0011
		interval 2>b	F(1,37) = 18.333, p = 0.0011
	gender	F(1,37) = 18.371, p = 0.0001	females<males
SBF_max_	interval	F (2,64) = 14.297, p = 0.0001	
		interval 1<b	F(1, 32) = 17.754, p = 0.0011
		interval 2<b	F(1,32) = 11.975, p = 0.022
	hypnotizability	F(1,32) = 6.491, p = 0.016	*highs<lows*

intervals: b (basal before CPT and CPT+AN), interval 1 (CPT_1_ and CPT+AN_1_), interval 2 (CPT_2_ and CPT+AN_2_); conditions : CPT, CPT+AN.

### Autonomic Variables

**Figure 4:**
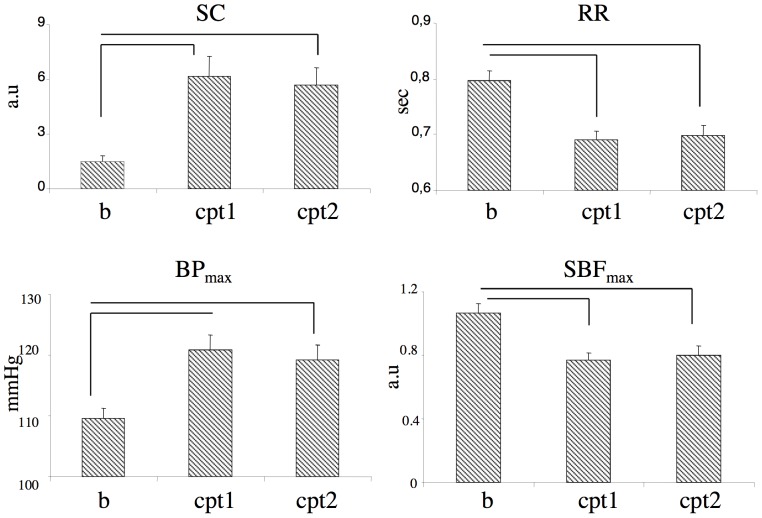
Autonomic changes. Skin conductance (SC) and systolic blood pressure (BP_max_) increase, while RR distance (RR) and skin blood flow (SBF_max_) decrease during cold pressor test. Variables are shown in the pooled conditions of presence (CPT+AN_1_, CPT+AN_2_) and absence of suggestions of analgesia (CPT_1_, CPT_2_) with respect to basal conditions (b) independently of hypnotizability. Lines, significant differences between conditions.

With respect to basal conditions, in both groups RR ( = 1/heart rate) and skin blood flow decreased significantly, while skin conductance and the systolic blood pressure increased significantly during immersion with and without suggestions of analgesia (Table 1, Figure 4). Only RR was modulated by suggestions, and was significantly longer in their presence. No significant interaction between hypnotisability and conditions was found for blood pressure, skin blood flow and skin conductance.

BP_max_ was significantly higher in males than in females and SBF_max_ was significantly lower in *highs* than in *lows* independently of conditions (Table 1).

## Discussion

The main findings of the present study were: a) the responsiveness of both *highs* and *lows* to non hypnotic suggestions of analgesia, b) the identification of distinct hypnotisability-related strategies of pain control, c) the observation of the similar cardiac and respiratory responses to suggestions of analgesia in *highs* and *lows,* in spite of their different subjective experience, d) the evidence that non hypnotic suggestions influence the parasympathetic, but not the sympathetic activity.

### Hypnotisability-related Pain Control Strategies

Results indicate that the pain experience elicited by cold pressor test undergoes hypnotisability-related control models.

The ability of cognitive inhibition, considered greater in *highs,* although not unanimously [34], [35], could likely account for the longer time of immersion observed in these subjects independently of suggestions [30]. Suggestions increased the time of immersion in both groups, but reduced pain intensity and increased pain thresholds only in *highs*. Moreover, cpt duration was positively correlated with pain threshold in *highs* and with pain tolerance in *lows* in both the absence and the presence of suggestions. In their absence, in *highs* it was also positively correlated also with pain tolerance.

We may hypothesize that these distinct psychophysical responses are associated with different cognitive strategies. In particular, the *highs’* pain modulation is in line with the observation of mechanisms acting on the sensory or affective dimensions of pain depending on the nature of suggestions [36], [37], which in the present study include both focused analgesia and relaxation. At variance, the modulation observed in *lows* (not involving pain intensity and threshold) could be mainly sustained by mechanisms responsible for emotional appraisal [38] and reward [39], as occurs in the general population, in which motivating and coping instructions increase tolerance, but do not alter pain perception [40].

### Respiratory and Autonomic Findings

The present study shows that the suggestions of analgesia elicit similar changes in the cardiovascular response to cold pressor test in *highs* and *lows,* independently of their subjective experience. This observation is in line with the findings obtained in the general population which had shown that the heart rate response to cold pressor test may be unrelated to pain ratings [41]. A similar dissociation between the subjective experience and the autonomic state had been observed in hypnotized *highs* during suggestions for emotional numbing modulating the autonomic activity in the absence of changes in the conscious experience [42], and possibly contributing to a later modification of the subjective experience [1], [2].

At variance with the reports on hypnotic analgesia in *highs,* who showed sympathetically-mediated reduction in vascular peripheral resistance [11], in the present study non hypnotic suggestions did not reduce the increase in systolic blood pressure and skin conductance as well as the decrease in skin blood flow elicited by cold pressor test. Since heart rate and the systolic blood pressure are controlled by both sympathetic and parasympathetic mechanisms, whereas skin conductance and skin blood flow are controlled only by the sympathetic branch of the autonomic system, we hypothesize that the subjects’ cardiac response to non hypnotic suggestions was substantially mediated by the parasympathetic activity.

The absence of cpt induced respiratory modulation is in line with other reports [12], [13], the lower skin blood flow observed in *highs* with respect to *lows* throughout the experimental session may be a consequence of the *highs’* greater absorption (TAS scores) and/or imagery abilities [8], [44] likely able to induce pain and peripheral vasoconstriction through imagery of the expected cold pressor test. Finally, the lower blood pressure observed in females with respect to males is in line with the current literature on gender related control of blood pressure [43] and the increased skin conductance in the non immersed hand of both groups should be interpreted as a general stress response [45].

### General Observations

The study has some limitations. One is the lack of assessment of a few psychological features potentially influencing the response to nociceptive stimulation. Information on pain expectancy and motivation to pain relief was not collected because completing questionnaires during the experimental session would have induced artefacts in the autonomic signals, evaluation of cognitive inhibition was too time consuming for participants. Moreover, in further experiments medium hypnotizable participants should be enrolled. Finally, we are aware that thresholds assessment may be influenced by individual factors limiting the reliability of the pain tolerance computed as the difference between cpt duration and pain threshold, although thresholds are commonly used in pain psychophysics.

However, our findings clearly show that both *highs* and *lows* respond to non hypnotic suggestions for analgesia during cold pressor test, that distinct pain control models can be hypothesized for the two groups, and that the cardiac correlates of pain modulation are likely to depend on the parasympathetic activity. The novelty and relevance of the present findings consist of the indication that, in healthy subjects, non hypnotic suggestions for analgesia are effective not only in *highs* and *medium*s, but also in *lows*. Thus, our findings extend the potential use of the suggestions of analgesia to the general population and may account for their efficacy in chronic pain patients independently of hypnotisability [46], [47], [48].
